# RNA as the stone guest of protein aggregation

**DOI:** 10.1093/nar/gkaa822

**Published:** 2020-10-17

**Authors:** Alexandra Louka, Elsa Zacco, Piero Andrea Temussi, Gian Gaetano Tartaglia, Annalisa Pastore

**Affiliations:** UK Dementia Research Institute at the Maurice Wohl Institute of King's College London, London SE5 9RT, UK; Center for Human Technologies, Central RNA laboratory, Istituto Italiano di Tecnologia, Genova 16152, Italy; UK Dementia Research Institute at the Maurice Wohl Institute of King's College London, London SE5 9RT, UK; University “Federico II’’ Napoli, via Cynthia, Napoli 80100, Italy; Center for Human Technologies, Central RNA laboratory, Istituto Italiano di Tecnologia, Genova 16152, Italy; Centre for Genomic Regulation (CRG), The Barcelona Institute of Science and Technology, Barcelona 08003, Spain and ICREA, 23 Passeig Lluıs Companys, Barcelona 08010, Spain; Charles Darwin department of Biology and Biotechnology, Sapienza University of Rome, Piazzale A. Moro 5, Rome 00185, Italy; UK Dementia Research Institute at the Maurice Wohl Institute of King's College London, London SE5 9RT, UK

## Abstract

The study of prions as infectious aggregates dates several decades. From its original formulation, the definition of a prion has progressively changed to the point that many aggregation-prone proteins are now considered *bona fide* prions. RNA molecules, not included in the original ‘protein-only hypothesis’, are also being recognized as important factors contributing to the ‘prion behaviour’, that implies the transmissibility of an aberrant fold. In particular, an association has recently emerged between aggregation and the assembly of prion-like proteins in RNA-rich complexes, associated with both physiological and pathological events. Here, we discuss the historical rising of the concept of prion-like domains, their relation to RNA and their role in protein aggregation. As a paradigmatic example, we present the case study of TDP-43, an RNA-binding prion-like protein associated with amyotrophic lateral sclerosis. Through this example, we demonstrate how the current definition of prions has incorporated quite different concepts making the meaning of the term richer and more stimulating. An important message that emerges from our analysis is the dual role of RNA in protein aggregation, making RNA, that has been considered for many years a ‘silent presence’ or the ‘stone guest’ of protein aggregation, an important component of the process.

## INTRODUCTION

This review deals with the concept of prion-like proteins and/or domains. This concept dates the 90s when Wickner found proteins in yeast with a behaviour similar to that observed for vertebrate prions (1). The definition was then transferred and widened to incorporate also proteins with properties somewhat different from those originally set for prions. The most recent interpretation of the prion-like term includes many different aggregation-prone proteins that are also often involved in the phenomenon of liquid-liquid phase separation, a concept of great actuality.

In our review, we retrace the concept of prion-like proteins to understand its meaning, applications and role in normal and pathologic functions. We explore the concept of prion-like proteins and their link to RNA-binding properties and liquid-liquid phase separation. The view that comes out from our analysis is the richness of meanings that the prion-like term has now incorporated with a direct link to essential metabolic pathways that, when altered, may directly lead to pathology. Using the paradigmatic example of the TDP-43 protein, we discuss the link between prion-like proteins and RNA, a component previously banned from the definition of prions. We could thus say that RNA has acted in prion and prion-like proteins as the ‘stone guest’ of the Mozart's opera Don Giovanni. This metaphorical expression indicates an impending presence (person or object) known by all but not explicitly acknowledged, that is invisible, silent and, consequently, rather disturbing and unpredictable. Accordingly, we discuss in this review how the role of RNA is not unique: we show how it can be both beneficial or detrimental for aggregation depending on the sequence and composition of RNA. Keeping this message in mind, we suggest that RNA could be used as a powerful way to interfere with protein self-assembly or aggregation if the synthax of protein–RNA interactions was fully decoded.

### The prion concept

It may be useful for our discussion to briefly trace back the origin of the prion concept. The term prion was originally used to describe a proteinaceous particle (PrP) with infectious properties that would exclusively consist of a single protein without the involvement of a nucleic acid genome (2). The concept originated from studies of animal and human diseases, scrapie in sheep, kuru and Creutzfeldt-Jakob diseases in humans, and spongiform encephalopathy in cows (mad cow disease) (3–6). The idea made great rumor at the time because it was noticed that all previously known pathogens, such as bacteria, parasites and viruses, are able to reproduce themselves through a genetic code and thus through nucleic acids. Stanley Prusiner showed instead that the scrapie infectious agent was susceptible to all agents that disrupt proteins, i.e. proteases, inactivation by chemical modification, chaotropic salts, urea (2). The hypothesis was also consistent with the resistance of the scrapie agent to ionizing and UV irradiation, extreme heat, high pressure and nucleases that act on nucleic acids, inactivating viruses and bacteria (2,3,7,8). In 1985, Prusiner and Charles Weissman discovered that a host cellular gene encoded the major protein found in purified preparations of the scrapie agent, and that the infectious material lacked the gene encoding the cellular prion protein (PrP^C^) (9). In the same year, Bruce Chesebro and Richard Race showed that PrP_27–30_, a protease-resistant fragment of infectious PrP (residues 80–231), is a normal component of both infected and uninfected mouse and hamster brain tissues (10,11). In 1993, Prusiner suggested that the scrapie infectious agent may be an immunologically inert host encoded protein (PrP^C^) that could misfold into a pathologic form (PrP^Sc^), by a conformational change of α-helices into a β-rich structure prone to undergo a liquid-to-solid phase transition, which is often called in the field ‘protein aggregation’. These β-rich aggregates were supposed to have structural features similar to those of ‘amyloids’ found in proteins associated with numerous other protein misfolding disorders (12–14). Amyloid fibrils are ordered protein aggregates, which assemble to form insoluble fibers resistant to degradation and composed predominantly of a β-sheet structure aligned perpendicularly to the fibril axis (cross-β conformation). The conversion would template the misfold of other natively folded PrP^C^ and cause a prion disease in susceptible hosts (15). Thus, two different but interconnected aspects are what makes prions a prion: the capacity to have a conformational change toward a β-rich structure (conformational switch) and the ability to transmit misfolding in a process that could be described as infectious (16).

### A conformational switch

Vertebrate prions are the very benchmark of the concept of a conformational switch. The conversion of PrP^C^ into the β-rich (ca. 47% β-sheet content) PrP^Sc^ is considered the key event in the transmission of prion disease or, in other words, of prion infectivity (17,18). The cellular form of mature human PrP^C^ comprises 231 residues after deletion of a 22-residue signal peptide (19). It is a diglycosylated protein, anchored to the outer leaflets of plasma membranes via glycosylphosphatidylinositol (GPI) although non-glycosylated and mono-glycosylated isoforms may co-exist (20,21).

**Figure 1.**
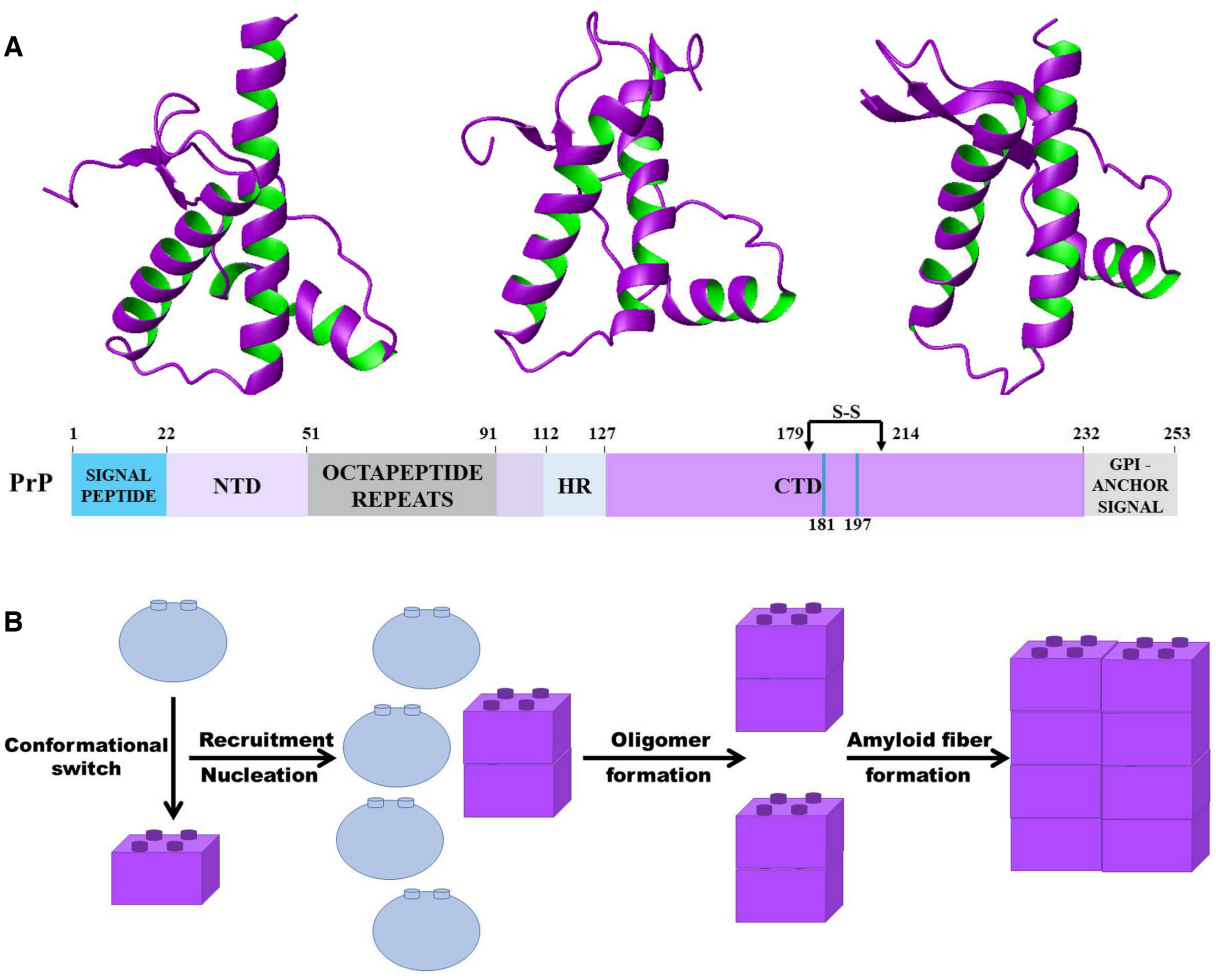
The structure of the prion protein and model of its aggregation. (**A**) Schematic architecture of the sequence and representative structures of the globular domain of PrP^C^, shown as ribbon models. From left to right: cow (pdb id: 1dwz), mouse (pdb id: 1ag2), and xenopous (pdb id: 1xu0). (**B**) Model of prion aggregation based on the templating properties of PrP^Sc^.

The prion sequence is composed of a intrinsically unstructured N-terminus and a globular C-terminus (22,23). The first structure of the C-terminal domain of a vertebrate PrP^C^ was that of the mouse protein solved by NMR spectroscopy in the Wuethrich's laboratory (24). Many other structures from the most diverse animals have been solved since (22,23). They all have similar folds consisting of three α-helices (H1, H2 and H3) and a short anti-parallel β-sheet and reflecting the remarkable degree of sequence conservation observed throughout evolution (Figure 1A).

The hypothesis behind PrP^Sc^ propagation is that the misfolded protein may create a tightly interdigitated steric zipper with the β-sheet of PrP^C^ (25,26) inducing the misfold of the correctly folded protein (Figure 1B). The structure of PrP^sc^ has not been proven beyond doubt (27). Only theoretical models are currently available and mostly deduced for the C-terminal domain (18). The main models are a triangular β-helix surrounded by C-terminal helices, proposed by the Prusiner's laboratory (28), another β-helix suggested by MD simulations consisting in a two-rung model of a left-handed β-helix (29) and a structure in which H2 and H3 of PrP^C^ are both converted in a parallel all-β architecture (30). These models have a high β-content but little else in common. A prominent role in attempts to find the structure of PrPsc has been recently played by cryo-EM studies (31,32). Even if a high-resolution structure of PrP^Sc^ remains to be solved, cryo-electron microscopy data now support a variety of other approaches in favor of the structure of PrP^Sc^ as a four-rung β-solenoid (32).

One interesting consideration is that most of the attention has remained focused on the structured globular C-terminus (22,33). This choice was partially dictated by the fact that it seemed easier to deal with a globular region of known and well-defined structure to reason about a conformational switch and to explain crossing species barriers. Nevertheless, the highly positive charged intrinsically unstructured N-terminus may have an important role in protein aggregation. Recently, it was reported that the N- and C-terminal regions may interact, thus disfavouring PrP aggregation (34,35). RNA might interfere with this intramolecular docking, although the relative strength of the interaction would play a decisive role.

### The role of RNA in prion conversion

The essentiality of the PrP^C^ to PrP^Sc^ structural conversion is also at the base of the original ‘protein-only’ hypothesis according to which the infectious component of the prion disease would consist solely of proteins without any nucleic acid element (36). However, prion deposition and toxicity profoundly differ among tissues, suggesting that other factors rather than solely PrP^C^ abundance may intervene in dictating prion accumulation and spread (37,38). Accordingly, *in vitro* protein-only aggregates display non-significant levels of infectivity (39). This evidence led to the suggestion that an additional unknown factor (a ‘protein X’ in Prusiner's words) could influence the PrP^C^ to PrP^Sc^ conversion (40). The investigation of this phenomenon led to the hypothesis that PrP^C^ aggregation into infectious PrP^Sc^ could be prompted by nucleic acids, which could act as catalysts for the propagation reaction (41–43) and restore latent infectivity (44). In particular, the idea that non-coding RNA (ncRNA) may have a regulatory function on other biomacromolecules brought to the investigation of the potential role that RNAs encoded by the host may play in the PrP^C^-to-PrP^Sc^ conversion during species transmission (43,45). There was however a substantial difference: nucleic acids would not transport genetic information but act as chaperones to lower the free energy barrier between PrP^C^ and PrP^Sc^, thus favoring conversion (46,47).

RNA is known to be able to either promote (48) or prevent (49) protein aggregation. Whether different sequences and structures of RNA could cause different effects on prion aggregation has been long debated (50,51). Some researchers also suggested that RNA could be at the basis of the various prion species (strains), either by promoting a liquid-to-solid phase separation or maintaining oligomers in a soluble phase-separated form (50,52,53). In other cases, it was reported that RNA, irrespective of structure and sequence, could affect both extent and rate of PrP aggregation, according to its concentration (54). Noticeably, all RNAs bind selectively to the PrP N-terminus, the protein low complexity domain (55,56). This is hardly surprising given the strong net positive charge of this region (22).

It is interesting to note that, in the end, nucleic acids came back to the prion equation in one way or the other. The intervention of RNA in acquiring PrP^Sc^ infectivity appears now necessary, although the functional and pathogenic nature of the RNA involvement remains unclear. This link between RNA-binding and the PrP intrinsically unfolded N-terminus is also interesting in light of what we shall say about prion-like sequences.

### The concept of prion-like proteins

A few years after Prusiner (2) put forward the protein-only hypothesis for scrapie, Wickner (1) proposed that protein conformational switches could be responsible for inheritance of phenotypes also in yeast. The first characterized yeast prions were propagating amyloid forms of the *Saccharomyces cerevisiae* proteins Sup35p and Ure2p, independently named [PSI] and [URE3] (57). The term prion was then extended to include proteins from other organisms and endowed with properties not necessarily directly linked to prions and with no sequence similarities with either yeast or vertebrate prions. This is why the term prion-like was introduced (58). Bona fide examples of effective delivery of infectious particles during cell division were demonstrated in the laboratories of Weissman (59) and Lindquist (60).

In the attempt to classify prion-like proteins, two conditions were defined as important: a high degree of intrinsic disorder and low complexity sequence regions enriched in asparagine, glutamine, tyrosine and glycine residues (61,62). This compositional bias would promote the formation of several local weak interactions that would promote misfolding. Interestingly, glutamines and asparagines were found to have an opposite effect on prion formation: asparagines promote benign prion formation, whereas a glutamine excess can lead to toxic non-amyloid aggregates (63). This signature based on unstructured regions with a bias in sequence composition towards low-complexity regions immediately informed the development of software able to predict prion-like regions in proteins.

Several bioinformatic studies have helped to identify prion-like domains. In a pioneering search in *S. cerevisiae*, the Lindquist group conducted a genome-wide bioinformatics survey using a hidden Markov sequence model to identify putative candidates on the basis of their compositional similarity to known prion forming domains that were validated experimentally (64). Many more programs were then developed and have predicted prions in all life domains (65), including viruses (66,67) and bacteria (68). Synthetic design of prions (69) indicated that the prion propensity is strongly linked to the aggregation propensity that can be predicted with methods to compute the kinetics of amyloid fibrils (70).

The compositional content is not an absolute rule: the [HET-s] prion from the fungus P. anserina (71), for instance, is not glutamine/asparagine-rich nor are the highly conserved vertebrate prions (72–74). Thus, in the attempt to cover as many cases as possible an alternative model, pWALTZ, was formulated which suggests a preferential nucleation promoted by a short amyloidogenic stretch able to trigger the amyloid conversion of the complete prion protein (16). The amyloid propensities of the stretch would in turn be modulated by the structural context.

This redefinition led to the inclusion in the prion-like term of numerous proteins associated with the formation of β-rich structures formed as a consequence of a conformational switch. It is often assumed that this switch is similar in all protein aggregation diseases and corresponds to a change from other conformations (disordered, helical, mixed) to a β-rich structure (13,14), even though the actual specific structures may vary widely.

### Prion-like RNA-binding proteins in neurodegeneration

**Figure 2.**
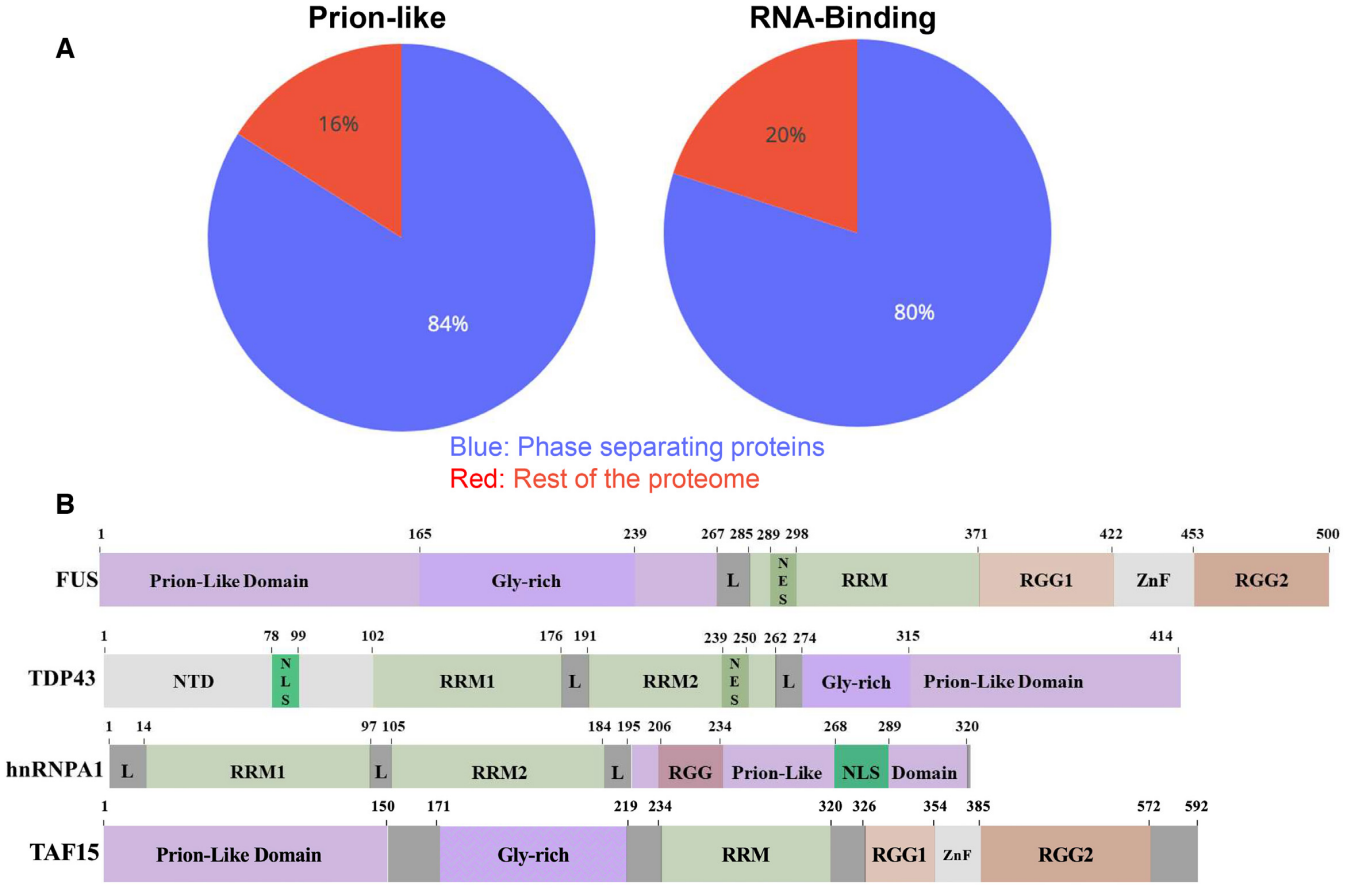
How the subsets of prion-like, RNA-binding and phase-separating proteins superpose. (**A**) Comparison of the sets of prion proteins, RBPs and proteins involved in phase separation shows a remarkable overlap of the three sets proteome-wide. Phase separating proteins are simultaneously more likely to comprise also the sets of prion-like domains and RBPs. Fraction of prion-like domains as defined in (90) and RBPs are calculated with respect to proteins found in stress granules, using rest of the proteome as a control. The enrichments, computed with Fisher exact test, are highly significant (*P*-values < 0.001) (81). (**B**) Block diagrams of prion-like proteins linked to neurodegeneration.

The use of software able to search prion-like regions was particularly helpful in finding functional correlations amongst prion-like sequences. Gene ontology (GO) annotations indicated that ∼30% of human proteins with prion-like domains function in RNA binding (70) and ∼33% function in DNA binding (75). Prion-like domains were found in ∼240 human RNA- or DNA-binding proteins and are known to have essential functions in mammals (76–78). Many of these proteins also contain RNA recognition motifs (RRM) (75,79). RRM-containing genes represent only ∼1% of the human protein-coding genome, but they comprise >10% of all genes containing prion-like domains (80). When the ability of proteins to aggregate in phase-separated coacervates was investigated, a co-occurrence of prion-like and RNA-binding domains was observed (81) (Figure 2A). The best known example of phase-separated assemblies are stress granules (SGs) (82), which form in the cytoplasm upon different physical and chemical insults and include several proteins such as FUS, TDP-43 and TAF15 (83).

Interestingly, it was also noticed that many of RNA-binding prion-like proteins are associated to neurodegenerative disorders (75,84). The association began with the identification of a trinucleotide repeat expansion in the gene encoding ataxin-1 that leads to a polyglutamine protein product and causes spinocerebellar ataxia type-1 (85). Since this discovery, several other human RNA-binding proteins with prion-like domains associated with disease were identified including, for instance, FUS, TDP-43 and TAF15 that are linked to amyotrophic lateral sclerosis (ALS) and frontotemporal dementia (FTD) (86) (Figure 2B). Recently, Maharana *et al.* (2018) (87) explicitly suggested that prion-like RNA-binding proteins like TDP43 and FUS are kept soluble in the nucleus by the ‘buffering’ action of high RNA concentrations. These links indicate that human proteins with prion-like domains are prone to deleterious misfolding events that underpin neurodegenerative disease.

### Prion-like proteins in liquid–liquid phase separation

RNA-binding prion-like proteins have more recently been linked to processes other than toxicity, such as the formation of SGs or other non-pathologic coacervates that are involved in the formation of membraneless organelles (82,88). This is reasonable since a compositional bias towards partially hydrophilic residues, together with a low representation of bulky hydrophobic ones, would help to reach the delicate balance between a protein being soluble but still able to form aggregates in given conditions.

The observation that proteins with prion-like domains are also RNA-binding suggests a potential link between these two motifs and their function (81). Tartaglia and coworkers, for instance, recently evaluated the co-presence of experimentally reported RNA-binding proteins (89) and prion-like motifs (90) in a well-known dataset of biological coacervates. These authors focused on one of the best known membraneless organelles, the SGs of *S. cerevisiae* (91) to analyse data from Jain *et al.* (82). They observed a >5-fold significant enrichment of RNA-binding proteins and prion-like domains in proteins associated to SGs as compared to the overall yeast proteome (81). It was thus hypothesized that the two motifs could cooperate and be present at the same time in SGs and other phase-separated assemblies. The cooperation would ensure formation of transient aggregates able to have storage and protective functions toward RNA.

Proteins that contain both RNA-binding and prion-like domains were also quantified both in the human and yeast proteomes and compared to proteins that contain only one or none of the two domains (81). In this analysis, the Prion-Like Amino Acid Composition (PLAAC) algorithm was used which searches protein sequences to identify probable prion-like regions using a hidden-Markov model (HMM) algorithm (64). The Fisher test indicated a significant co-occurrence in both proteomes (*P*-values < 0.001) thus supporting a possible cooperation between these domains in the phase separation process. Finally, it was hypothesized that the self-interaction capacity of prion-like domains can lead to solid-like aggregation in given conditions. As a large poly-anionic molecule involved in the maintenance of fluidity, RNA could counterbalance the effect of prion-like domains, shifting the equilibrium towards a more dynamic organization (92). Disordered regions are also known to interact with RNAs (89,93). Thus, it could be hypothesized the intriguing possibility that the disordered prion-like domains establish weak, transient interactions with RNA, especially in the context of an actively regulated coacervate. While the prion domain has a clear role in promoting protein interactions and assembling condensates, RNA binding could influence the final state. The fine interplay between protein and RNA interactions regulates the formation of membrane-less organelles, inducing quick formation of ribonucloprotein assemblies and promoting their fast disaggregation (81).

### The case study of TDP-43

Let us now analyze in closer details how these concepts apply to one of the best studied prion-like proteins, TDP-43, taken as an informative example. TDP-43 is an essential protein that plays an important role in mRNA splicing, degradation, stabilization, translation and transportation (94,95). TDP-43 aggregates are observed in the neurons of ALS and FTD patients. In these pathologies, the protein mis-localizes from the nucleus where it normally resides to the cytoplasm where it forms inclusions (96–98). Whether TDP-43 aggregates are the direct cause of cytotoxicity in FTD and ALS, or simply accumulate as a response to other pathogenic events is still debated (99,100), although increasing evidence supports a direct toxic effect (98).

**Figure 3.**
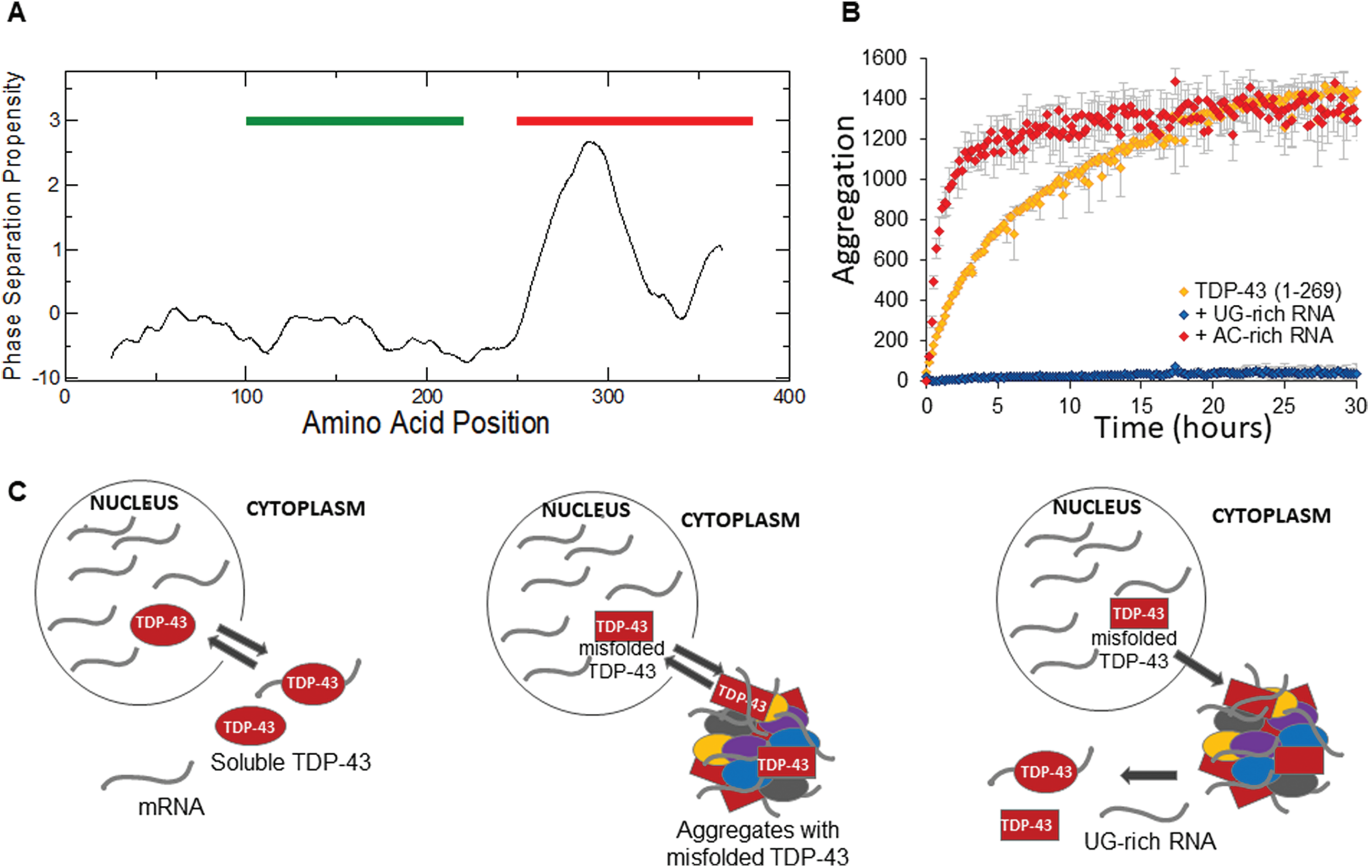
Effects of RNA binding on the solubility of TDP-43. (**A**) catGRANULE identifies a region promoting phase separation in the prion-like domain of TDP-43 (red line; RNA-binding domains are marked in green; the profile is scaled ×10). (**B**) Kinetics of aggregation of N-RRM1–2 in the absence and presence of a UG-rich and a AC-rich RNA aptamer (at equimolar stoichiometries) as reported in Zacco *et al.* (2019). The kinetics were represented proportionately to the variation of the emission fluorescence of ProteoStat™ as a function of time. (**C**) Model of how ALS would be triggered. **Left panel:** In the nucleus, wild type TDP-43 is kept in its native soluble form by its interactions with nucleic acids. It temporarely shuffles to the cytosol to release the bound mRNA and it is stable enough to relocate within the nucleus. **Middle panel:** When mutations that directly compromise its RNA-binding ability or drastically alter its structure occur, TDP-43 can aggregate in permanent occlusions, such as stress granules, together with other proteins and RNAs. **Right panel:** The presence of UG-rich RNA/TG-rich DNA, natural binding partners of TDP-43, may be able to stabilise the protein native structure, preventing or disruption aberrant aggregation.

The structure of TDP-43 comprises of an N-terminal domain linked by an unstructured region to two RRM repeats, that are followed by an intrinsically unstructured C-terminus. The RRM motifs recognize preferentially UG or TG-rich RNA sequences (101). A relatively short glycine rich prion-like stretch (342–366) within the C-terminus was considered for a long time the ‘minimal aggregative segment’ in that is capable of recapitulating some of the aggregation properties of the whole protein (102). This region has a strong propensity to promote the aggregation of TDP43 in phase-separated coacervates, as predicted with the *cat*GRANULE software (Figure 3A) (103). The program predicts phase-separation propensity on the basis of physico-chemical properties such as structural disorder, nucleic acid binding propensity and amino acid composition (e.g. arginine-glycine and phenylalanine-glycine enrichment) (81). The prion-like domain is not required for RNA- or DNA-binding activity, but is critical for alternative splicing of some mRNAs and for protein-protein interactions (104) The importance of this region for protein aggregation was reinforced by the observation that many of the ALS- and FTD-linked TDP-43 mutations lie in or near the prion-like domain. Several of them promote immediate TDP-43 aggregation and enhance proteotoxicity.

We recently proved that, contrary to what previously postulated, other regions of TDP-43 are prone to misfolding and promote the formation of aggregates also in the absence of the prion-like domain (105,106): constructs containing the two RRM domains or a fragment truncated just after RRM2 form aggregates with amyloid features. More in general this lesson tells us that aggregation is often not under the control of only one region and that modular proteins may often contribute with multiple aggregation-prone regions (88).

We reasoned that, according to its role as an RNA-binding protein, aggregation of TDP-43 could be modulated by RNA. This view was comforted by several considerations. We had previously shown that native interactions could be used as a powerful and specific means to inhibit protein aggregation (107). Other authors had also already demonstrated that aggregation of TDP-43 could be influenced by DNA/RNA binding even though the precise role of nucleic acids was controversial: some studies had indeed provided evidence that TDP-43 binding to cognates DNA or RNA sequences could prevent aggregation (108,109) and that binding of TDP-43 to the 3' UTR of its cognate mRNA enhanced its solubility (110,111). Other reports claimed instead that RNA induces TDP-43 fragments to adopt highly toxic misfolded conformations (84). We have shown instead that RNA aptamers have a strong influence on aggregation but the effect is not uniform (106): We proved that, when incubated with UG-rich sequences, the *in vitro* aggregation of the TDP-43 construct 1–269 (comprising the N-terminus up to the end of RRM2) was abrogated already at equimolar protein:RNA ratio; instead, the presence of non-UG-rich RNA sequences could induce faster aggregation of the same protein fragment (Figure 3B). This means that aptamers that share compositional and sequence similarities with natural partners and that have a high affinity for the protein, have a strong inhibitory effect against aggregation. Conversely, aptamers with no resemblance to the native partners and low affinity binding can in fact increase aggregation. This behaviour fits very well with the hypothesis that RNA may direct prion-like RNA-binding proteins either towards maintenance of their native structure or towards a faster conformational switch, according to its sequence and structure. RNA may therefore be the element that renders the proteins more soluble or prone to aberrant aggregation.

To support these results, an increasing number of TDP-43 mutations are being identified in regions other than the prion-like. Among these, the most interesting ones are Lys181Glu and Lys263Glu mutants that affect two residues directly implicated in RNA binding (112). These mutations appreciably reduce the RNA-binding affinity. We found that while constructs containing the RRM domains of the wild-type and mutant proteins have similar aggregation properties *in vitro*, aggregates are readily generated with the mutated protein because RNA is unable to contrast protein aggregation.

Together these considerations may help us to understand the mechanisms going on in the triggering of ALS and how any misregulation of RNA binding may affect TDP-43 aggregation (Figure 3C). They also suggest a possible new line of therapeutic intervention: If we could design RNA aptamers able to bind to TDP-43 tightly enough to interfere with aggregation but not with the native function we could use these sequences as the bases for lead-compounds able to halt disease progression.

## CONCLUSIONS

**Figure 4.**
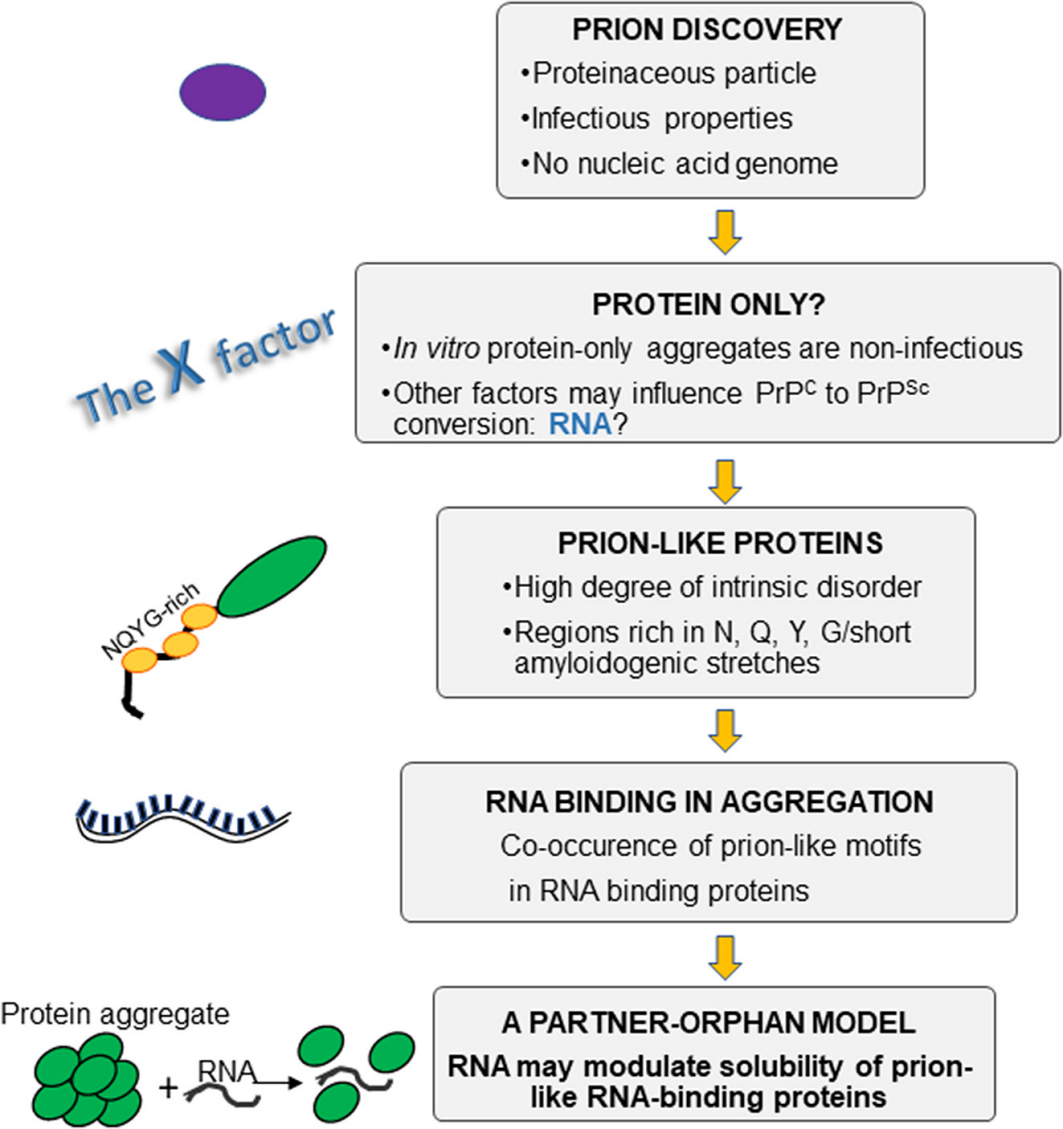
Flowchart of the milestones of the prion and prion-like concept.

In summary, we have given here a historical perspective of prion-like proteins (Figure 4). We have discussed how the concept of prions has evolved and covers now almost all examples of amyloid-prone proteins, extending from TDP-43 and FUS to cover also other macromolecules involved in protein aggregation such as Aβ, α-synuclein and polyglutamine containing proteins, which have not been covered in this review (16). It is interesting to notice how RNA, at first banned from the prion concept and remained for many years the Stone Guest of protein aggregation, has finally been ‘rehabilitated’: it is now established that RNA plays an active role both in neurodegenerative diseases and in important cellular functions (113). Its role is however not unique and this stands out as an important lesson: RNA can be both beneficial or detrimental depending on its sequence and composition. From an evolutionary point of view, this implies an exquisite evolutionary fine-tuning and co-evolution of RNA, proteins and their functional requirements that deserves future close attention. It is interesting to note that Docter *et al.* (114) have hypothesized that organisms may use RNA as a molecular chaperone to prevent protein unfolding and aggregation. We also propose to reconsider the mechanisms that promote protein aggregation and include a new ‘partner-orphan’ model for prion-like RNA binding proteins in which their solubility may be determined by the presence or absence of RNA. This new concept, reminiscent of previous work on proteins (115–117), would provide to the cell a powerful means to allow formation of membraneless organelles or to prevent aggregation depending on the RNA sequence and composition. This perspective also proposes a new strategy towards the development of anti-aggregation drugs based on RNA aptamers and sheds new light onto the physical forces that determine protein aggregation.
